# Infection prevention and control perspective and practices among healthcare workers in Bangladesh: A multicenter cross section

**DOI:** 10.1017/ash.2023.326

**Published:** 2023-09-29

**Authors:** Md. Golam Dostogir Harun, Shariful Amin Sumon, Aninda Rahman, Md Mahabub Ul Anwar, Md. Saiful Islam

## Abstract

**Background:** Infection prevention and control (IPC) is a critical feature of preventing the spread of healthcare-associated infections (HAIs) in hospitals. IPC practices are particularly important in resource-constrained and crowded hospital settings. The successful implementation of infection prevention measures depends on healthcare worker (HCW) knowledge of, attitude toward, and practice (KAP) of IPC. In this project, we assessed the KAP of HCWs and identified factors associated with IPC compliance at tertiary-care hospitals in Bangladesh. **Methods:** From September 2020 to January 2021, we conducted this hospital-based cross-sectional assessment at 11 tertiary-care hospitals. A semistructured questionnaire was used to conduct face-to-face interviews with physicians, nurses, and cleaning staff who were directly involved in patient care. Based on >75% of the total score, each KAP component was divided into adequate knowledge, favorable attitude, and safe practice. We performed descriptive analysis and multivariate logistic regression to determine the KAP score and associated factors influencing IPC compliance in hospital settings. **Results:** In total, 1,728 HCWs were interviewed; 76.8% of the participants had adequate knowledge on IPC and 54.6% reported safe practices. However, only 16.2% of HCWs had a favorable attitude toward IPC. Among the 3 HCW groups, nurses had the highest KAP scores (76.07±12.7) followed by physicians (69.8±16.2), and cleaning staff (34.4±27.3). Only 29.2% of HCWs reported having received IPC training, and they cited heavy workload as a barrier to IPC guideline adherence. HCWs having adequate knowledge showed 9 times higher odds of safe IPC practice (AOR, 9.36; 95% CI, 5.47–16.04). HCWs who had a favorable attitude toward IPC were 16 times as likely to perform safe practice toward IPC activities (AOR, 15.5; 95% CI, 10.27–23.42). **Conclusions:** Knowledge of safe practices and having a favorable attitude toward IPC are key components of a successful IPC program. Significant improvements are required among all levels of HCWs in Bangladesh tertiary-care hospitals, especially cleaning staff. Educational interventions to train on IPC guidelines, plus monitoring, could improve HCW safe practices.

**Figure:**
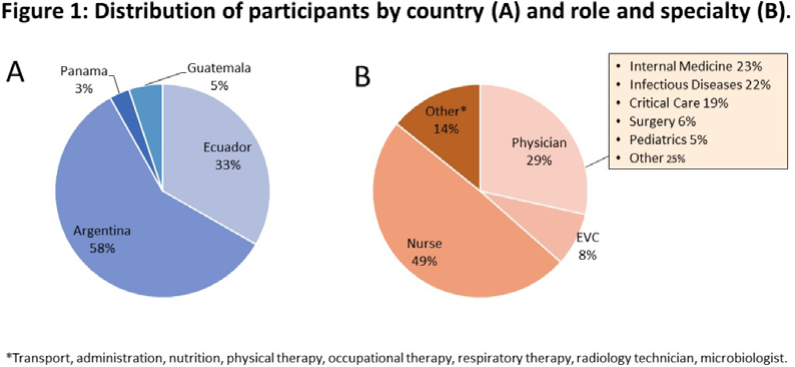

**Figure:**
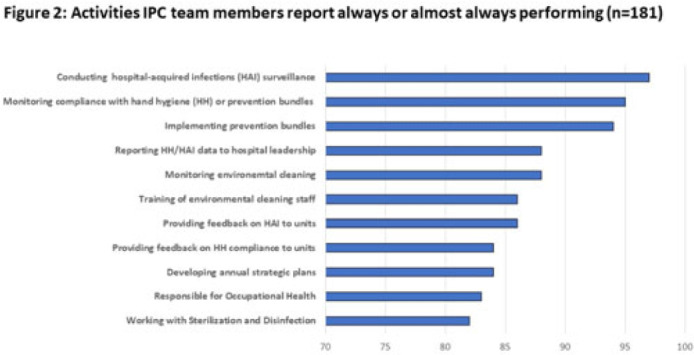

**Disclosures:** None

